# Optical Coherence Tomography Angiography Vessel Density Changes after Acute Intraocular Pressure Elevation

**DOI:** 10.1038/s41598-018-24520-x

**Published:** 2018-04-16

**Authors:** Qi Zhang, Jost B. Jonas, Qian Wang, Szy Yann Chan, Liang Xu, Wen Bin Wei, Ya Xing Wang

**Affiliations:** 1 Beijing Institute of Ophthalmology, Beijing Tongren Hospital, Capital Medical University, Beijing Ophthalmology and Visual Sciences Key Laboratory, Beijing, China; 2 Beijing Tongren Eye Center, Beijing Tongren Hospital, Capital Medical University, Beijing Ophthalmology and Visual Science Key Lab, Beijing, China; 3 Department of Ophthalmology, Medical Faculty Mannheim of the Ruprecht-Karls-University Heidelberg, Seegartenklinik, Heidelberg, Germany

## Abstract

To investigate changes in retinal vessel density in optic nerve head (ONH) and macula after acute intraocular pressure (IOP) elevation, we conducted a prospective observational study. Eyes with IOP rise ≥5 mmHg after 2-hour dark room prone provocative test (DRPPT) were included. Vasculature of ONH and macula was examined by optical coherence tomography angiography (OCTA) at baseline and after DRPPT. Among the 65 eyes of 42 individuals, 40 eyes with qualified images were enrolled. Mean IOP rise was 9.6 ± 4.2 mmHg (5.0–23.3 mmHg) after DRPPT. Retinal vessel density did not differ after IOP rise for either the papillary region (optic nerve head and radial peripapillary capillary layer) or the macula region (superficial, deep and outer retinal layer) (*P* > 0.05). Vessel density in each subregion did not change either. If only enrolled eyes with IOP rise ≥10 mmHg, similar results were obtained in condition of IOP increase by 15.0 ± 3.6 mmHg. To conclude, eyes with an acute IOP elevation by 10 or 15 mmHg for two hours, while the blood pressure remained constant, the vessel density in both ONH and macula region examined by OCTA did not show significant changes. The observations fit with an IOP-related autoregulation in retinal blood flow for a moderate elevation of IOP.

## Introduction

The blood perfusion of the optic nerve head (ONH) and retina depends on the local arterial blood pressure and the intraocular pressure (IOP) as counter-pressure against the arterial blood pressure. Subsequently, the mean ocular perfusion pressure has been defined as the difference of 2/3 of the mean blood pressure minus IOP, with mean blood pressure calculated as diastolic blood pressure +1/3 of the blood pressure amplitude (i.e., systolic blood pressure minus diastolic blood pressure)^1^. Correspondingly, marked elevation of IOP to suprasystolic levels leads to a stop of retinal blood circulation and a temporary central retinal artery occlusion. Investigations revealed that a moderate elevation in IOP or moderate changes in blood pressure did not markedly affect the retinal blood circulation, a phenomenon which was called retinal autoregulation^2–9^. Studies suggested that the retinal autoregulation may be impeded in open-angle glaucoma fitting with the notion of a vascular element in the pathogenesis of glaucomatous optic neuropathy^10–14^.

In the previous investigations, the retinal blood perfusion was examined applying techniques such as fluorescein angiography, confocal scanning laser ophthalmoscopic angiography with fluorescein dye, color Doppler imaging, Canon laser blood flowmetry, scanning laser Doppler flowmetry, and retinal photographic oximetry^15–18^. All these techniques had disadvantages such as limitations in spatial resolution, relatively low validity, or only indirectly assessing parameters of blood flow, to mention a few. Split-spectrum amplitude-decorrelation angiography (SSADA) associated with optical coherence tomography angiography (OCTA) is a new method that can visualize the vascular networks in separate layers of the retina in the macular region and in the ONH^19–21^. Recent studies confirmed a high intra-visit repeatability and inter-visit reproducibility of the measurements taken with OCTA, a high spatial resolution of the images, and the possibility of the OCTA technique to assess for the first time the retinal blood circulation system at different levels of the retina and ONH^22^. In view of these new technical possibilities, we conducted the present study to re-assess the relationship between the blood circulation in the retina and optic nerve in eyes at a normal IOP and at an elevated level of IOP. In contrast to previous investigations in which artificial means, such as a suction cup, were applied to increase the IOP, we used the physiological model of IOP elevation by performing a dark room provocative test^23–25^.

## Methods

The prospective comparative study included all individuals who routinely and consecutively underwent a dark room prone provocative test (DRPPT) from November 2015 through January 2016 and who showed an elevation in IOP of more than 5 mmHg during the DRPPT. The Ethical Review Committee of the Beijing Tongren Hospital approved the study and confirmed its adherence to the provisions of the Declaration of Helsinki for research involving human subjects. Informed consent was obtained from each participant after providing an explanation of the nature and possible consequences of the study. Exclusion criteria were an age <18 years, any optic nerve disease including glaucomatous optic neuropathy, any eye disease that might affect the quality of fundus images, and difficulties in fixation.

The DRPPT has routinely been performed in the Beijing Tongren Hospital for the examination of individuals suspected for acute primary angle closure, which was defined as patients with narrow but not occluded anterior angles but otherwise normal. The details of the test were described previously^22–24^. Shortly before the test and at one and two hours after the start of the test, IOP was measured by noncontact tonometry (Topcon CT-60; Topcon Ltd, Tokyo, Japan). All tonometric measurements were performed three times, and the mean value of the three measurements was used for further statistical analysis. Before the start of the DRPPT and within 5 minutes after the end of the test, the vasculature of ONH and macula was visualized by OCTA. We used the RTVue XR Avanti device (ReVue software, version 2014.2.0.93; Optovue Inc, Fremont, California, USA) with the Angio Retina mode (3 × 3 mm^2^) and the Angio Disc (4.5 × 4.5 mm^2^) mode. Ocular biometry (optical low-coherence reflectometry; Lensstar 900 Optical Biometer; Haag-Streit, Koeniz, Switzerland) was additionally carried out after the DRPPT.

**Figure 1:**
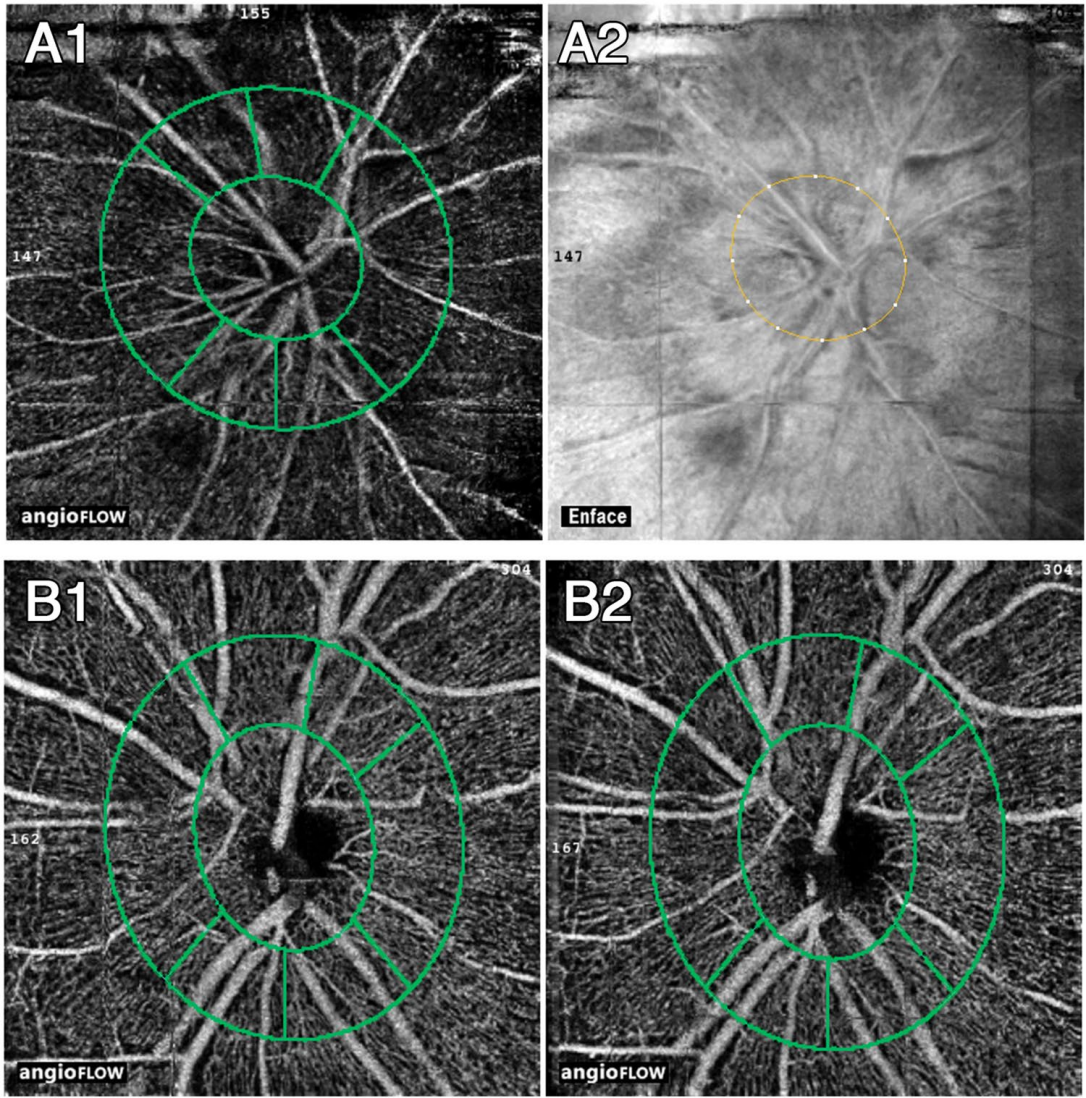
Illustration of images with insufficient quality and therefore dismissed. An eye excluded due to poor signal strength index (SSI = 44) was shown in **A1**. And its en-face image shown in **A2**. Images of an eye with obvious offset deviation so as to be dismissed were shown in **B1**. And **B2**. The position of the optic disc shifted and subregions were not consistent from baseline (**B1**.) to the post dark room prone provocative test (**B2**.).

Criteria for inclusion into the study were an IOP rise during the DRPPT of ≥5 mmHg and the availability of OCT angiographic images of sufficient quality. Insufficient quality of the OCT angiographic images was defined by a signal strength index lower than 50 or by an obvious offset deviation (Fig. 1). The examinations were performed by a trained ophthalmologist (QZ) in a masked manner without knowledge of results of other examinations.

**Figure 2:**
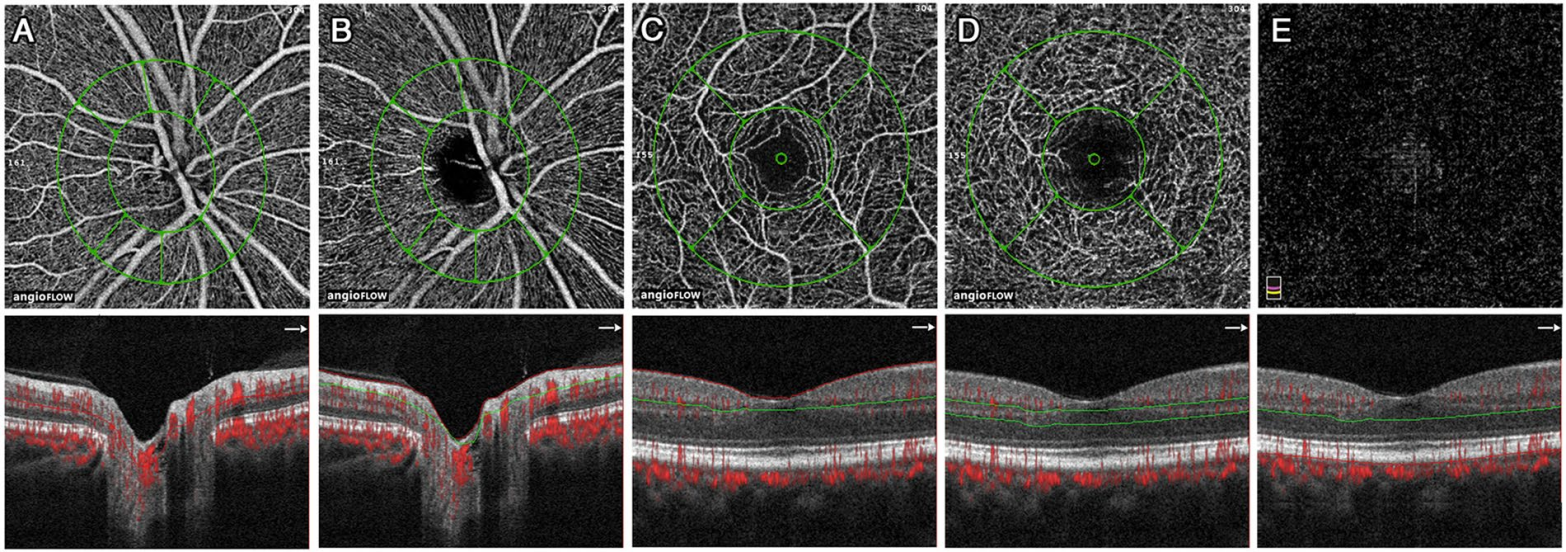
Optical coherence tomographic angiographic image of the papillary region and macular region with measurement of the blood flow density. The vessel density measurement in the papillary region were composed of the measurement at the optic nerve head (ONH) layer and measurement at the radial peripapillary capillary (RPC) layer in the papillary region of 4.5 × 4.5 mm^2^ area, and the density at the superficial retinal layer, the deep retinal layer, and the outer retinal layer in the macular region of 3 × 3 mm^2^ area. The papillary region was divided into an intrapapillary region and into six peripapillary regions (nasal, inferonasal, inferotemporal, temporal, superotemporal, superonasal) located between an inner ring (defined by the of Bruch’s membrane opening) and an outer ring with a radius 0.75 mm larger than the radius of the inner ring. The macular region was divided into a foveal area and four parafoveal areas (temporal, superior, nasal, inferior) between two concentric circles of 1 mm diameter and 2.5 mm diameter, respectively. **(A**) The ONH layer (from the inner limiting membrane to 150 μm below the inner limiting membrane); (**B**) the RPC layer (from the upper boundary of the inner limiting membrane to the lower boundary of the nerve fiber layer); (**C**) the superficial retinal layer (from approximately 3 μm below the inner limiting membrane to 15 μm below the inner plexiform layer); (**D**) the deep retinal layer (from 15 μm below the inner plexiform layer to 70 μm below the inner plexiform layer); **(E**) the outer layer (from 70 μm below the inner plexiform layer to 30 μm below the retina pigment epithelium layer).

The quantitative ocular vessel density was automatically measured by the built-in software of the OCT device. The vessel density measurement in the papillary region (4.5 × 4.5 mm^2^) were composed of the measurement at the ONH layer and measurement at the radial peripapillary capillary (RPC) layer, which extended from the inner limiting membrane to 150 μm below the inner limiting membrane, and from the upper boundary of the inner limiting membrane to the lower boundary of the nerve fiber layer, respectively. The measurement of capillary density in the macula region (3 × 3 mm^2^) were composed of the density at the superficial retinal layer, the deep retinal layer, and the outer retinal layer, which extended from approximately 3 μm below the inner limiting membrane to 15 μm below the inner plexiform layer, from 15 μm below the inner plexiform layer to 70 μm below the inner plexiform layer, and from 70 μm below the inner plexiform layer to 30 μm below the retina pigment epithelium layer, respectively^19^. The optic disc region was divided into an intrapapillary region and six peripapillary regions, while the macula region was divided into a fovea region and four parafoveal areas (Fig. 2).

In another study composed of 55 patients undergoing the DRPPT, the arterial blood pressure was evaluated before and after the DRPPT and no change was found in both systolic and diastolic blood pressure (Data not shown).

Statistical analysis was performed using a statistical software package (SPSS for Windows, version 22.0; IBM-SPSS, Chicago, IL). Measurements obtained before and after the DRPPT were compared with each other using the paired Student t test. All measurements were described as mean ± standard error. All *P*-values were based on 2-sided tests and considered statistically significant if less than 0.05.

## Results

The study included 65 eyes of 42 individuals suspect for occludable angles with otherwise normal eyes, who showed an IOP rise ≥5 mmHg after the dark room prone provocative test (DRPPT). There were 32 women and 10 men included with age of 56.4 ± 9.2 years (range: 41 to 76 years). The IOP increased from 17.3 ± 3.5 mmHg to 26.9 ± 6.4 mmHg, with the mean rise of 9.7 ± 5.4 mmHg.

After excluding the OCTA images with insufficient quality defined by a signal strength index lower than 50 (6 eyes) and those with obvious offset deviations (9 eyes), a total of 40 eyes of 29 individuals (21 women) were included in the final analysis. The mean age was 55.2 ± 7.2 years (range: 42–69 years) and mean axial length was 22.9 ± 1.1 mm (range: 21.2–25.5 mm). The mean anterior chamber depth was 2.1 ± 0.3 mm (range: 1.5–2.8) and mean lens thickness was 4.7 ± 0.3 mm (range: 3.9–5.3 mm). The IOP increased from 17.2 ± 2.9 mmHg (range: 10–26 mmHg) at baseline to 26.4 ± 5.6 mmHg (range: 19–49 mmHg) at the end of DRPPT, with a mean rise of 9.6 ± 4.2 mmHg (range: 5.0–23.3 mmHg).

The vessel density in the ONH region measured at baseline of the study did not differ significantly from the measurements obtained at the end of the DRPPT, either at the ONH layer (58.1 ± 3.3% versus 58.5 ± 3.1%; *P* = 0.14) or at the RPC layer (56.3 ± 4.6% versus 56.5 ± 4.2%; *P* = 0.44) (Table 1). The measurements taken in its subregions including the intrapapillary region and separate peripapillary sectors did not differ between the baseline readings and the readings obtained at the end of the DRPPT (Table 1).

**Table 1:** Blood Vessel Density (BVD) (Mean ± Standard Deviation; %) at Baseline and at the End of the Dark Room Prone Provocative Test (DRPPT) in the optic disc region.

	BVD at Optic Nerve Head Layer			BVD at Radial Peripapillary Capillary Layer		
	Baseline (Mean ± SD)%	End of DRPPT (Mean ± SD)%	*P-*Value	Baseline (Mean ± SD)%	End of DRPPT (Mean ± SD)%	*P-*Value
Whole area (4.5 × 4.5 mm^2^)	58.1 ± 3.3	58.5 ± 3.1	0.135	56.3 ± 4.6	56.5 ± 4.2	0.436
Inside disc region	53.4 ± 5.4	53.8 ± 5.1	0.151	41.5 ± 10.2	41.6 ± 10.2	0.651
Peripapillary region						
Nasal	61.4 ± 4.0	61.6 ± 4.3	0.651	61.3 ± 4.8	61.7 ± 5.0	0.412
Inferonasal	63.5 ± 5.2	64.5 ± 4.3	0.169	64.6 ± 5.9	65.2 ± 5.2	0.437
Inferotemporal	64.7 ± 4.4	64.5 ± 5.7	0.744	67.5 ± 5.5	65.2 ± 5.2	0.449
Superotemporal	64.1 ± 5.4	64.3 ± 5.3	0.612	67.3 ± 5.9	67.5 ± 5.6	0.756
Superonasal	62.2 ± 4.9	62.3 ± 4.6	0.813	62.9 ± 5.7	62.8 ± 4.8	0.830
Temporal	61.5 ± 4.3	62.0 ± 3.4	0.319	64.8 ± 4.3	65.1 ± 4.4	0.341
Whole	62.4 ± 3.5	62.7 ± 3.3	0.317	64.1 ± 4.3	64.3 ± 4.2	0.533

The vessel density in the macular region at baseline and at the end of DRPPT didn’t differ either at the three layers (superficial retinal layer: 51.6 ± 2.3% versus 51.3 ± 2.6%, *P* = 0.42; deep retinal layer: 55.8 ± 2.2% versus 55.8 ± 2.4%, *P* = 0.80; outer retinal layer: 24.2 ± 5.2% versus 23.8 ± 5.7, P = 0.66) (Table 2). Again all vessel density measurements taken in subregions of the macula neither differed between the baseline readings and the readings taken at the end of the test (Table 2).

**Table 2:** Blood Vessel Density (BVD) (Mean ± Standard Deviation; %) at Baseline and at the End of the Dark Room Prone Provocative Test (DRPPT) in the Macular Region.

	BVD at superficial retinal layer			BVD at deep retinal layer			BVD at outer retinal layer		
	Baseline (Mean ± SD)%	End of DRPPT (Mean ± SD)%	*P*-Value	Baseline (Mean ± SD)%	End of DRPPT (Mean ± SD)%	*P*-Value	Baseline (Mean ± SD)%	End of DRPPT (Mean ± SD)%	*P*-Value
Whole area (3 × 3 mm^2^)	51.6 ± 2.3	51.3 ± 2.6	0.419	55.8 ± 2.2	55.8 ± 2.4	0.799	24.2 ± 5.2	23.8 ± 5.7	0.660
Fovea region	25.7 ± 5.3	25.3 ± 5.1	0.299	21.8 ± 5.4	21.8 ± 5.8	0.964			
Para Fovea									
Temporal	52.4 ± 2.3	52.1 ± 2.5	0.523	57.0 ± 3.0	56.7 ± 3.7	0.612			
Superior	54.1 ± 3.0	54.0 ± 2.9	0.865	59.9 ± 2.7	59.8 ± 2.8	0.776			
Nasal	52.3 ± 2.5	52.3 ± 3.1	0.994	57.1 ± 3.7	57.0 ± 4.1	0.762			
Inferior	54.4 ± 2.9	53.7 ± 3.7	0.168	60.0 ± 3.2	60.0 ± 2.8	0.980			
Whole	53.3 ± 2.3	53.0 ± 2.6	0.484	58.5 ± 2.8	58.4 ± 2.9	0.720			

If only eyes with an IOP rise of more than 10 mmHg were enrolled (12 eyes of 8 individuals; mean IOP rise: 15.0 ± 3.6 mmHg), vessel density in the papillary area and the macula area did not change significantly during the test period (Table 3).

**Table 3:** Blood Vessel Density (BVD) (Mean ± Standard Deviation; %) at Baseline and at the End of the Dark Room Prone Provocative Test (DRPPT) in the Optic Nerve Head Region and in the Macular Region in Eyes with an Intraocular Pressure Elevation of >10 mmHg (mean rise 15.0 ± 3.6 mmHg) during the DRPPT.

			Baseline (Mean ± SD) %	End of DRPPT (Mean ± SD) %	*P-*Value
BFD in the Optic Nerve Head Region	Whole Area	ONH layer	57.4 ± 7.1	57.4 ± 4.0	0.898
		RPC Layer	54.7 ± 7.1	55.0 ± 6.2	0.720
	Intrapapillary Region	ONH layer	51.3 ± 8.2	51.4 ± 7.6	0.772
		RPC Layer	36.9 ± 13.6	36.9 ± 12.7	0.997
	Peripapillary Region	ONH layer	61.3 ± 5.2	61.5 ± 4.7	0.819
		RPC Layer	62.6 ± 6.8	63.1 ± 6.3	0.601
BFD in the Macular Region	Whole Area	Superficial Layer	51.0 ± 3.1	50.1 ± 3.0	0.301
		Deep Layer	54.9 ± 2.6	54.3 ± 2.4	0.371
		Outer Layer	21.9 ± 5.4	21.7 ± 7.8	0.913
	Foveal Region	Superficial Layer	26.5 ± 4.2	25.7 ± 3.7	0.309
		Deep Layer	23.0 ± 3.2	22.4 ± 2.3	0.510
	Parafoveal Region	Superficial Layer	52.6 ± 2.6	51.7 ± 2.6	0.284
		Deep Layer	57.0 ± 3.3	56.3 ± 3.0	0.365

In the final step, one random eye per subject was selected for analysis. In the 29 eyes from 29 participants, similar results were obtained that the vessel density in both the papillary area and the macula area did not change significantly while IOP increased by 9.1 ± 4.4 mmHg after the DRPPT (Table 4).

**Table 4:** Blood Vessel Density (BVD) (Mean ± Standard Deviation; %) at Baseline and at the End of the Dark Room Prone Provocative Test (DRPPT) in the Optic Nerve Head Region and in the Macular Region in group of one eye per subject enrolled (mean rise 9.1 ± 4.4 mmHg) during the DRPPT.

			Baseline (Mean ± SD) %	End of DRPPT (Mean ± SD) %	*P-*Value
BFD in the Optic Nerve Head Region	Whole Area	ONH layer	57.7 ± 3.6	58.4 ± 3.4	0.080
		RPC Layer	56.2 ± 5.2	56.3 ± 4.7	0.813
	Intrapapillary Region	ONH layer	52.9 ± 6.0	53.4 ± 5.7	0.265
		RPC Layer	41.4 ± 11.4	41.4 ± 11.2	0.953
	Peripapillary Region	ONH layer	62.1 ± 3.9	62.8 ± 3.6	0.078
		RPC Layer	64.0 ± 4.8	64.1 ± 4.4	0.734
BFD in the Macular Region	Whole Area	Superficial Layer	51.0 ± 2.3	50.8 ± 2.7	0.656
		Deep Layer	55.3 ± 2.1	55.5 ± 2.3	0.446
		Outer Layer	24.5 ± 5.1	24.0 ± 6.2	0.566
	Foveal Region	Superficial Layer	24.4 ± 4.4	24.1 ± 4.3	0.543
		Deep Layer	20.8 ± 3.7	20.8 ± 4.2	0.948
	Parafoveal Region	Superficial Layer	52.9 ± 2.4	52.6 ± 2.8	0.591
		Deep Layer	58.1 ± 3.0	58.0 ± 3.0	0.935

## Discussion

The results of our observational study suggest that either the papillary or the macular vessel density as measured by OCTA did not change markedly during a mean physiologic rise in IOP of about 10 mm Hg to a mean maximum of 26.4 ± 5.6 mmHg (range: 19–49 mmHg) for a duration of two hours. The findings support the notion of an autoregulation of the blood flow in the ONH and in the macula during a rise in IOP ranging between 10 mmHg and 26 mmHg for a period of two hours. To our knowledge, this is the first time that the autoregulation was observed in human retinal microcirculation in condition of natural IOP elevation.

The results obtained in our study were consistent with the findings that the autoregulation of the blood flow reported in previous studies on retinal autoregulation mainly based on the techniques of the laser Doppler flowmetry^3,6,26–29^. Riva CE and colleagues found that the blood flow in the optic nerve region remained constant down to a perfusion pressure of approximately 22 mmHg by suction cup^3^. In a recent study by Schmidl and colleagues, the authors observed a wide inter-individual variability of the response of the ONH blood flow to a change in ocular perfusion pressure in healthy subjects and identified a subgroup of individuals with abnormal autoregulation of the ONH blood flow^6^. In an investigation by Boltz and coworkers, the regulation of the ONH blood flow using laser Doppler flowmetry was examined during a separate increase in IOP by the suction method and increase in arterial pressure by an isometric exercise^7,26^. During the handgrip-induced increase in ocular perfusion pressure by approximately 15 mmHg ONH blood flow did not change, indicating that the blood flow of the ONH vasculature reduced potentially by vasoconstriction. Conway and coworkers assessed the blood flow responses before and after application of a microkeratome for refractive corneal surgery^27^. After elevating the IOP to a level of higher than 85 mmHg for 90 seconds in 10 eyes, no difference in ocular-perfusion measurements were detected by color Doppler imaging and the Heidelberg retinal flowmeter between the baseline measurements and the readings obtained shortly after the IOP elevation. It was appropriate for quantitative defining the blood flow in larger vessels with Doppler technique, but not accurate in the capillaries. Comparing with OCTA, the laser Doppler flowmetry, and the laser speckle flowgraphy can only observe within a limited area of the retina, and the inter-visit variability was reported to be not ideal, with 12.8% and 6.6% to 21.2%, for the laser speckle flowgraphy and for the laser Doppler flowmetry, respectively^30,31^.

Recently there were several studies showing the vessel density change after IOP decrease quantified by OCTA. Holló G found that the peripapillary angioflow density increased at 2 to 4 weeks after medical reduction of IOP of more than 50% in 6 eyes of 4 glaucoma patients^32^. Zéboulon and colleagues observed the retinal vessel density one month after surgically induced IOP lowering of 44.2 ± 4.8% in 21 eyes out of 21 glaucoma patients however didn’t find distinct changes comparing with baseline parameters^33^. Shin JW found an improvement in peripapillary retinal microcirculation in glaucoma patients 3 months after trabeculectomy. The difference in the results may perhaps be associated with the level of the IOP change, the methods of IOP decrease, age of patients, and the level of glaucomatous damage. It will be interesting to compare the microvascular changes between glaucoma patients and normal in the same study settings, thus to furtherly investigate the vascular factors of glaucoma pathogenesis^34^.

Animal studies have also focused on the blood flow regulation in normal and experimental glaucoma^35–38^. Using OCT based microangiography, Zhi and colleagues examined the retinal capillary filling in rats with an acutely elevated IOP^35^. They observed that the retinal blood flow diminished linearly with increasing IOP when the increase in IOP was 30 mmHg to 100 mmHg. Wang *et al*. found that the ONH blood flow remained at a constant level within a range of ocular perfusion pressure of 41 mm Hg and above, with IOP elevation by laser treatment to the trabecular meshwork in monkeys^36^. Shibata *et al*. showed that ONH blood flow in l-2-aminoadipic acid injected rabbit eyes was significantly decreased with a reduction of OPP during IOP elevation to 50 and 70 mm Hg^37^.

The RPC is a superficial layer of capillaries with relative constant caliber, paralleling with the retinal nerve fiber layer in the peripapillary region. One of its functions is likely to play a role in supplying nutrition to the retinal ganglion cells^39^. With the unique pattern and distribution of the vessels, RPC were thought to be vulnerable to the increase in IOP comparing with other retinal capillaries, thus possibly be involved in the pathogenesis of glaucoma^40,41^. According to our study, the blood flow remained unchanged in the RPC layer after moderate IOP rise in 2 hours, showing similar regulation resisting pressure with other layers in the retina. It is speculated that the potential link between the RPC and glaucoma does not necessarily lie in its susceptibility to the IOP.

The mostly unchanged blood flow in the macular region of the retina and in the ONH region at different IOP levels contrasts with a IOP rise related morphological changes in the choroid and the ONH. Previous studies revealed that eyes with an IOP rise in the DRPPT showed a IOP-rise dependent thinning of the choroid, a thinning of the neuroretinal rim, in particular at the temporal border of the optic disc, a centrifugal shift of the retinal pigment epithelium on parapapillary Bruch’s membrane, and a condensation of the tissue covering the lamina cribrosa at the bottom of the optic cup^23–25^.

Limitations of our study should be discussed. First, arterial blood pressure was not measured in the specific 42 participants of our study. However, another group of 55 randomly-selected patients with similar settings showed that with acute IOP rise after the 2-hour-DRPPT, both the systolic and the diastolic blood pressure didn’t change from baseline of the test. Second, our study included only a selected group of individuals who were at risk for a rise in IOP provoked by the dark room test. It has remained open, whether the results of our study can be generalized to any individual. Third, absence of prove is no prove of absence, so that potential differences between the measurements might have become statistically significant if the number of study participants had been increased. The differences between the measurements taken at baseline of the DRPPT and at the end of the test were however usually less 1% of the mean, and for the comparison of 12 parameters, measurements obtained at baseline of the test were higher than the measurements taken at the end of the DRPPT, and for the comparison of 15 parameters, the readings taken at the end of the test were higher than the measurements obtained at the start of the DRPPT (Tables 1 and 2). It may indicate that with a larger number of participants differences might not have become statistically significant. Fourth, the repeatability of OCTA measurements were not tested in the current study. Since the IOP after the DRPPT was time-sensitive and its normalization began to occur shortly, it was not possible to do the measurement of blood flow with the same IOP at 2 different time. Previous studies demonstrated a high intra-visit repeatability and inter-visit reproducibility of OCTA, including the own work of our group^22^. Fifth, since an abnormal autoregulation of retinal blood flow in open angle glaucoma was suggested in several studies^10–14^, it is important to compare the present results with glaucoma patients. Further work should be applied. A strength of our study is the application of OCTA as a new technique, the use of a physiological model of elevation of IOP, and the assessment of the blood flow in the macular region in addition to the blood flow characteristics in the ONH region.

In conclusion, healthy eyes with an acute and physiologic IOP elevation of 10 mmHg or 15 mmHg for two hours did not show significant changes in the capillary vessel density both of the ONH and macula as examined by OCTA, supporting the notion of an IOP-related autoregulation of the blood circulation system of the ONH and retina for a moderate elevation of IOP for a period of 2 hours.
